# Hypothalamic CaMKKβ mediates glucagon anorectic effect and its diet-induced resistance

**DOI:** 10.1016/j.molmet.2015.09.014

**Published:** 2015-10-22

**Authors:** Mar Quiñones, Omar Al-Massadi, Rosalía Gallego, Johan Fernø, Carlos Diéguez, Miguel López, Ruben Nogueiras

**Affiliations:** 1 Department of Physiology, CIMUS, University of Santiago de Compostela-Instituto de Investigación Sanitaria, Santiago de Compostela, 15782, Spain; 2 CIBER Fisiopatología de la Obesidad y Nutrición (CIBERobn), 15706, Spain; 3 Department of Morphological Sciences, School of Medicine, University of Santiago de Compostela-Instituto de Investigación Sanitaria, Santiago de Compostela, 15782, Spain; 4 Department of Clinical Science, K. G. Jebsen Center for Diabetes Research, University of Bergen, Bergen, Norway

**Keywords:** Food intake, Glucagon, Hypothalamus, PKA, CamKKβ

## Abstract

**Objective:**

Glucagon receptor antagonists and humanized glucagon antibodies are currently studied as promising therapies for obesity and type II diabetes. Among its variety of actions, glucagon reduces food intake, but the molecular mechanisms mediating this effect as well as glucagon resistance are totally unknown.

**Methods:**

Glucagon and adenoviral vectors were administered in specific hypothalamic nuclei of lean and diet-induced obese rats. The expression of neuropeptides controlling food intake was performed by *in situ* hybridization. The regulation of factors of the glucagon signaling pathway was assessed by western blot.

**Results:**

The central injection of glucagon decreased feeding through a hypothalamic pathway involving protein kinase A (PKA)/Ca^2+^-calmodulin-dependent protein kinase kinase β (CaMKKβ)/AMP-activated protein kinase (AMPK)-dependent mechanism. More specifically, the central injection of glucagon increases PKA activity and reduces protein levels of CaMKKβ and its downstream target phosphorylated AMPK in the hypothalamic arcuate nucleus (ARC). Consistently, central glucagon significantly decreased AgRP expression. Inhibition of PKA and genetic activation of AMPK in the ARC blocked glucagon-induced anorexia in lean rats. Genetic down-regulation of glucagon receptors in the ARC stimulates fasting-induced hyperphagia. Although glucagon was unable to decrease food intake in DIO rats, glucagon sensitivity was restored after inactivation of CaMKKβ, specifically in the ARC. Thus, glucagon decreases food intake acutely via PKA/CaMKKβ/AMPK dependent pathways in the ARC, and CaMKKβ mediates its obesity-induced hypothalamic resistance.

**Conclusions:**

This work reveals the molecular underpinnings by which glucagon controls feeding that may lead to a better understanding of disease states linked to anorexia and cachexia.

## Highlights

• Glucagon stimulates PKA and inhibits CaMKKβ and AMPK in the arcuate nucleus (ARC).
• Down-regulation of glucagon receptor in the ARC increases fasting-induced hyperphagia.
• Glucagon is unable to decrease food intake in diet-induced obese (DIO) rats.
• In DIO rats, glucagon fails to alter CaMKKβ and its downstream targets AMPK and pACC.
• Down-regulation of CaMKKβ in the ARC restores glucagon sensitivity in obese rodents.

## 1 Introduction

Glucagon, a pancreatic polypeptide essential for glucose homeostasis identified in the 1920s, is secreted by alpha cells in response to low blood glucose [1], [2]. This hormone counteracts hypoglycemia and opposes insulin actions by stimulating hepatic glucose synthesis and mobilization [3]. Several reports have also indicated that glucagon controls a plethora of other biological actions, including lipid metabolism, thermogenesis, energy expenditure and satiation [2], [4]. Indeed, glucagon receptor antagonists and humanized glucagon antibodies appear to be promising therapies for obesity and type II diabetes in preclinical trials [5], [6], [7]. However, recent research on glucagon has challenged our knowledge of the biological actions of this hormone. Elegant reports have demonstrated that in contrast to the hormone's stimulatory effect on the production of hepatic glucose when released into the circulation, glucose production in the liver is inhibited when glucagon acts in the hypothalamus [8], [9].

Glucagon also inhibits food intake in humans, a finding first shown more than five decades ago [10], [11]. Still, the molecular underpinnings of glucagon's anorectic action barely have been studied. Pancreatic glucagon seems to be involved in the production of postprandial satiety without causing aversive effects [12]. The injection of antibodies against glucagon at the onset of the first meal stimulated feeding [13] suggesting that glucagon was necessary for the physiological termination of meals. Pharmacological reports indicated that glucagon administered through the hepatic-portal vein inhibited feeding in control rats but not in vagotomized rats [14], whereas other reports indicated that its intraperitoneal administration increased food intake [15]. At the central level, glucagon binding sites have been found in the rat brain [16], and injection of glucagon directly into the CNS suppressed feeding to a much higher degree than that of peripheral administration [17]. On the other hand, in mice lacking the glucagon receptor, food intake and body weight were decreased when these mice were fed a high fat diet (HFD) [18], which may have been caused by elevated levels of glucagon-like peptide 1 (GLP-1). On the other hand, glucagon also plays a pathogenic role in diet-induced type 2 diabetes, as it is essential in different rodent models of hyperglycemia, and it is an enabler of the diet-induced hyperinsulinemia [19]. This is supported by the fact that metabolic manifestations of diabetes cannot occur without glucagon action [20].

In this study, we sought to investigate the specific hypothalamic sites and neuronal pathways by which central glucagon inhibits food intake. Furthermore, we also examined the central molecular underpinnings involved in DIO-induced glucagon resistance.

## 2 Material and methods

### 2.1 Animals and surgery

Eight to 10-week-old (250–300 g) male Sprague Dawley rats were housed in individual cages under conditions of controlled temperature (23 °C) and illumination (12-hour light/12-hour dark cycle). Diet-induced obese (DIO) rats were fed on HFD (60% fat content, Research Diets) for 16 weeks. Animals were killed by decapitation, and brains were removed and frozen immediately. All experiments and procedures involved in this study were reviewed and approved by the Ethics Committee of the USC, in accordance with EU normative for the use of experimental animals.

### 2.2 Implantation of intracerebroventricular cannulae and treatments

Rats were anesthetized by an intraperitoneal injection of ketamine (100 mg/kg body weight)/xylazine (15 mg/kg body weight) and intracerebroventricular cannulae were implanted as previously described [21], [22], [23] (see Supplementary information). Glucagon (120, 240, 480, 1000 ng/rat), PKA inhibitor, H-89 (62 ng/rat = 24 μM; Sigma Chemical, USA), PKA activator, Sp-cAMPS (90 ng/rat = 40 μM; Tocris Bioscience, St. Louis, MO), and a glucagon receptor antagonist, des-His^1^ (Glu^9^) glucagon amide (500 ng/rat; Tocris Bioscience, St. Louis, MO), were used. Glucagon was dissolved in 0.01 N-HCl, diluted with saline and adjusted to pH 7.4 as described previously [24].

### 2.3 Stereotaxic acute infusion of glucagon in specific hypothalamic nuclei

Specific hypothalamic nuclei cannulae were implanted stereotaxically in fed rats. We targeted the ARC and the ventromedial nucleus (VMH) bilaterally as previously reported [21], [22], [23]. The coordinates used to reach the ARC were anterior to bregma (AP): 2.85, lateral to the sagittal suture (L): ±0.3, and ventral from the surface of the skull (V): 10.2. The coordinates used the VMH were AP: 2.4/3.2, L: ±0.6, V: 10.1. The incision was closed with sutures and rats were kept warm until fully recovered.

### 2.4 Stereotaxic microinjection of adenoviral and lentiviral expression vectors

Adenoviral expression vectors containing the constitutively active (CA) form of AMPK (AMPKα-CA) (3.83 × 10^10^ PFU/mL) (Viraquest, North Liberty, IA), the dominant negative (DN) form of CAMKKβ (1.01 × 10^10^ PFU/mL) (SL115816; SignaGen Laboratories, Gaithersburg, MD), or GFP controls (3.0 × 10^10^ PFU/mL) and lentiviral vectors over-expressing shRNA against glucagon receptor or GFP (control) (shRNA glucagon receptor clone ID: TRCN0000028701; 4.0 × 10^7^ TU/mL, Sigma–Aldrich Inc., Buchs, Switzerland) were injected bilaterally into the ARC AP: 2.85 mm, L: ±0.3 mm, and V: 10.2 mm, with a 25-gauge needle (Hamilton), as previously reported [23], [25], [26].

### 2.5 Western blot analysis

Western blot was performed as previously described [21], [22], [23] (see Supplementary information). The antibodies used are indicated in Supplementary Table 1.

### 2.6 Immunohistochemistry and immunofluorescence

Rat brains were fixed by perfusion followed by immersion (12 h) in 10% buffered formalin for 24 h. Brains were cut into 50 μm thick slices using a Vibratome^®^ Series 1000. Detection of diaminobenzidine (DAB) immunohistochemistry, GFP immunofluorescence and fluorescein-isothio-cyanate (FITC) were performed as previously reported [21], [22] using a rabbit anti-glucagon receptor antibody (Abcam; Cambridge, UK).

### 2.7 *In situ* hybridization

*In situ* hybridization was performed as previously described [27], [28] (see Supplementary information).

### 2.8 Statistical analysis and data presentation

To calculate the sample size we used the “resource equation” method in order to calculate the “E” value for each individual experiment [29]. This method is based on ANOVA and it is applicable to all animal experiments. Any sample size, which keeps E between 10 and 20 should be considered adequate [29]. Data are expressed as mean ± SEM. mRNA and protein data were expressed in relation (%) to control (vehicle-treated) rats. Error bars represent SEM. Kolmogorov–Smirnov test (5 < n < 7) and Shapiro–Wilk test (n ≥ 7) were performed to test normality. When variables were normally distributed Student t test or ANOVA followed by post-hoc Bonferroni adjustment comparing groups versus control were performed. When variables were not normally distributed the nonparametric tests Mann–Whitney U test or Kruskal–Wallis test followed by Dunn's multiple comparisons test were performed. For multiple comparisons, each group was compared with the saline control group. *P* < 0.05 was considered significant.

## 3 Results

### 3.1 Short-term central administration of glucagon inhibits food intake

Single ICV injections of glucagon (120, 240, 480 and 1000 ng/rat, doses adapted from a previous study [17]) significantly decreased food intake in a dose-dependent manner, reaching statistical significance at 480 ng/rat. These effects diminished with time and after 6 h there was no longer any statistically significant effect (Figure 1A). ICV glucagon significantly decreased AgRP gene expression (Figure 1B), whereas NPY, CART and POMC remained unaltered after 1 h (Figure 1B and Supplemental Figure. 1). Glucagon activates PKA, and we measured phosphorylated cAMP response element-binding protein (pCREB) as a marker of PKA activity and glucagon efficiency [8]. We also assessed important regulators of feeding downstream of PKA, such as calcium/calmodulin-dependent protein kinase kinase beta (CaMKKβ), AMPK and enzymes involved in fatty acid synthesis [30], [31]. ICV glucagon increased hypothalamic protein levels of pCREB and decreased CaMKKβ (Figure 1C). CaMKKβ is upstream AMPK, and, phosphorylated levels of AMPK (pAMPK) and its target *phospho-acetyl-CoA carboxylase* (pACC) were also significantly decreased, whereas acetyl-CoA carboxylase (ACC) and *fatty acid synthase* (FAS) were up-regulated in ICV glucagon-treated rats in comparison to saline-treated rats when measuring proteins in whole hypothalamus lysates (Figure 1C).

### 3.2 The anorectic action of glucagon is physiologically relevant and is mediated by glucagon receptor

In order to corroborate the specificity of the anorectic action of glucagon, we used a well-established glucagon receptor antagonist, des-His1 (Glu9) glucagon amide [8]. ICV injection of the glucagon receptor antagonist at different doses (0.5, 1 and 2 μg/rat) increased food intake in a dose-dependent manner (Figure 2A). When we injected a sub-effective dose (0.5 μg/rat) of the glucagon receptor antagonist 20 min before ICV glucagon, the anorectic action of ICV glucagon was completely blunted (Figure 2B). In addition to this pharmacological finding, we next evaluated the physiological importance of glucagon receptor in the ARC to regulate feeding. For this, we used lentiviruses encoding shRNA against glucagon receptor or GFP (control) injected stereotaxically into the ARC [32]. Infection efficiency was assessed by immunostaining of GFP in the ARC (Figure 2C) and decreased protein levels of glucagon receptor in the ARC (Figure 2D). The genetic inhibition of glucagon receptor in the ARC of lean rats significantly increased fasting-induced hyperphagia (Figure 2E). Furthermore, we also tested that AgRP protein levels were significantly up-regulated after the genetic inhibition of glucagon receptor (Figure 2D). Overall, these results indicate that glucagon receptors within the ARC are required for the control of feeding at short-term.

### 3.3 Central protein kinase A regulates feeding and is essential for the anorectic effect of glucagon

It is well-established that the hepatic action of glucagon is mediated by PKA [33], therefore, we next investigated if PKA might be also involved in the anorectic action of glucagon in the CNS. First, we tested the hypothesis that direct activation of central PKA might decrease food intake. Using the specific PKA activator Sp-cAMPS (90 ng/rat dose) [8], we found significantly decreased food intake at 4, 6 and 8 h post-administration in rats fed a chow diet (Figure 3A). Furthermore, using the PKA inhibitor H-89 (62 ng/rat) [8] prior to ICV glucagon administration blunted the glucagon-induced hypophagia (Figure 3B) and blocked the inhibitory effect of glucagon on hypothalamic CaMKKβ, pAMPK and pACC (Figure 3C). Altogether, these results indicate that central PKA regulates feeding and it is essential for the anorexic effect of glucagon.

### 3.4 The anorectic action of glucagon is mediated by the inactivation of AMPK in the hypothalamic arcuate nucleus

Consistent with previous reports indicating that the glucagon receptor is located in the mediobasal hypothalamus [8], [16], we detected glucagon receptor immunoreactivity in the arcuate (ARC) and ventromedial (VMH) nuclei of rat hypothalami (Supplemental Figure. 2). Therefore, we next sought to investigate the hypothalamic area responsible for the anorectic action of glucagon. We specifically injected glucagon in the ARC and the VMH of rats. To assess the effects of glucagon in both hypothalamic nuclei, we performed a combined injection of vehicle or glucagon with fluorescein-isothio-cyanate (FITC) that allowed us to control the diffusion of the treatment within the hypothalamus. Our histological analyses showed no diffusion of FITC outside the targeted nuclei, indicating that both ARC and VMH injections were specific (Figure 4A,E). We also assessed the efficiency of the injection by increased protein levels of glucagon in the ARC and VMH nuclei (Figure 4B,F). The administration of glucagon into the ARC decreased food intake (Figure 4C), whereas its administration into the VMH did not modify feeding (Figure 4G). In line with the reduced food intake, the injection of glucagon into the ARC increased protein levels of pCREB and decreased CaMKKβ, pAMPK and pACC in the ARC (Figure 4D), whereas glucagon injection into the VMH decreased protein levels of pCREB but did not change the expression of the regulators downstream PKA (Figure 4H).

As these results indicated that ARC neurons were responsible for the glucagon-induced hypophagia, we next aimed to elucidate the contribution of AMPK activity in the ARC. To this end, we used adenoviruses encoding constitutively active forms of AMPK (AMPKα-CA) together with GFP or control adenovirus expressing GFP alone [22], [23], [25], injected stereotaxically into the ARC of rats ICV treated with vehicle or glucagon. The efficiency of the stereotaxic injections in the ARC was corroborated by immunostaining of GFP (Figure 4I). The administration of AMPKα-CA adenoviruses into the ARC was accompanied by reversed hypophagia in rats treated with central glucagon (Figure 4J). Consistently, the decreased levels of the downstream AMPK target pACC in the ARC of rats treated with ICV glucagon was also blunted when AMPK was activated in the ARC (Figure 4K).

### 3.5 DIO rats are resistant to ICV glucagon-induced hypophagia and their feeding remain unaltered after the down-regulation of glucagon receptor in the ARC

A recent study indicated that the hepatic actions of hypothalamic glucagon were disrupted in rats fed a HFD [8]. In this work, we evaluated the effect of ICV glucagon on food intake in rats fed a HFD during 16 weeks. As a positive control, we used an ICV dose of GLP-1 (10 μg/rat) that decreased food intake as expected (Figure 5A). However, the same dose of ICV glucagon (480 ng/rat) that was effective in rats fed a chow diet was unable to decrease food intake in rats fed a HFD (Figure 5A). Since these rats were heavier than lean animals, we also injected a higher dose of ICV glucagon (1 μg/rat), which remained ineffective (Figure 5A). In spite of the lack of effect on food intake, ICV glucagon increased hypothalamic protein levels of pCREB (Figure 5B). However, ICV glucagon did not alter hypothalamic protein levels of CAMKKβ, pAMPK and pACCα in DIO rats (Figure 5B). These data suggest that the impairment of the hypothalamic glucagon-induced signaling pathway in obese rats occurs downstream of PKA. To test this hypothesis, we first evaluated whether direct activation of hypothalamic PKA was able to decrease food intake in DIO rats. The same dose of ICV Sp-cAMPS (90 ng/rat dose) that was effective in rats fed a chow diet (Figure 3A) was unable to decrease food intake in rats fed a HFD (Figure 5C), corroborating that DIO rats have a disrupted hypothalamic glucagon signaling that mediates the anorectic action downstream PKA. In order to evaluate the importance of endogenous glucagon receptors to mediate food intake in DIO rats, we next used lentiviruses encoding shRNA against glucagon receptor or GFP (control) injected stereotaxically into the ARC [32]. Infection efficiency was assessed by immunostaining of GFP in the ARC (Figure 5D) and decreased protein levels of glucagon receptor in the ARC (Figure 5E). Contrary to the results obtained in lean rats, the genetic inhibition of glucagon receptor in the ARC did not modify fasting-induced hyperphagia in DIO rats (Figure 5F).

### 3.6 Inhibition of CaMKKβ in the ARC restores glucagon sensitivity in DIO rats

In order to elucidate the precise molecule responsible for DIO-induced glucagon resistance, we next assessed the contribution of CaMKKβ. We used adenoviruses encoding dominant negative (DN) CaMKKβ together with GFP or control adenovirus expressing GFP alone as previously described by other laboratories [34], [35]. First, we demonstrated that the inhibition of CaMKKβ activity in the ARC of lean rats significantly decreased fasting-induced hyperphagia (Supplemental Figure. 3). These data confirm that hypothalamic CaMKKβ is physiologically relevant for the control of feeding. We next inhibited CaMKKβ activity in the ARC of DIO rats treated with vehicle or ICV glucagon. Infection efficiency was assessed by immunostaining of GFP in the ARC (Figure 6A) and decreased protein levels of the downstream PKA target pACC in the ARC (Figure 6B). Similar to results shown in Figure 5A, ICV glucagon failed to decrease feeding in DIO rats. However, the administration of CaMKKβ-DN adenoviruses into the ARC restored the ICV glucagon-induced hypophagia in DIO rats (Figure 6C). Importantly, this anorectic effect of ICV glucagon in DIO rats was obtained at the same dose (480 ng/rat) that was effective in lean rats. These results showed that the obesity-induced hypothalamic resistance to the anorectic action of glucagon is mediated by CaMKKβ.

## 4 Discussion

Despite the importance of the α-cell and glucagon in the regulation of glycemia and nutrient homeostasis, the physiology of this cell type and hormone remains largely elusive. Recently, there has been resurgence in glucagon research because of reports that the combination of glucagon and GLP-1 reduces obesity [5], [36] and restores leptin responsiveness in obese mice [6]. Furthermore, hypothalamic glucagon inhibits hepatic glucose production [8], [9]. The anorectic action of glucagon is an issue that makes this hormone attractive as a pharmacological target to treat obesity [5], [6], [7]. Yet, its central site of action and the mechanisms involved have not been fully characterized.

The present experiments confirm that activation of central glucagon signaling acutely and transiently decreases food intake in rodents [17]. We describe for the first time a hitherto unrecognized mechanism of action of glucagon on the regulation of food intake via PKA-CaMKKβ-dependent pathways in the ARC. More specifically, we found that the central injection of glucagon increases hypothalamic levels of pCREB, indicating an activation of PKA, and decreases hypothalamic levels of CaMKKβ and its downstream targets pAMPK and pACC. The blockade of central PKA blunted the anorectic action of glucagon, consistent with the inability of glucagon to inhibit pAMPK when PKA was inactive. AMPK is a metabolic sensor responding to hormones and nutrient signals [31] and the inhibition of AMPK after PKA activation has previously been reported in hypothalamic cell lines [37]. A recent study showed that PKA activation leads to inhibition of AMPK also in human hepatic cells [38]. Similar to our results on food intake, the hypothalamic [8] and hepatic [39] activation of PKA is also necessary for the actions of glucagon in liver, suggesting that central PKA is an essential player for the actions of glucagon on feeding and glucose production.

Although the neural circuitries in the brain controlling the anorectic action of glucagon are unknown, glucagon binding sites are widely located in brain areas, including the hypothalamus [8], [16], a key center in the control of energy homeostasis. Our findings show that activation of central glucagon signaling decreases AgRP mRNA expression in the ARC, consistent with previous studies that have demonstrated co-localization of the glucagon receptor in AgRP neurons [8]. We focused our study on the ARC and the VMH, two nuclei located in the mediobasal hypothalamus. We found that specific injection of glucagon into the ARC, but not VMH, inhibited feeding. Consistently, the activation of AMPK specifically in the ARC was sufficient to block the anorectic action of this hormone. Thereby, our results indicate that the ARC plays a key role in the hypophagic action of glucagon. Importantly, this mechanism seems to be of physiological relevance since the down-regulation of glucagon receptors in the ARC stimulates fasting-induced hyperphagia. In this regard, it is interesting to point out that even though the down-regulation of the glucagon receptor in the ARC showed a moderate efficiency, it was sufficient to cause a clear transient effect on feeding, suggesting that the endogenous glucagon signaling plays an important role on feeding behavior in the short-term. Certainly, glucagon receptors are also located in extra-hypothalamic areas [16], and our results do not exclude the possibility that other brain sites might also be involved in the effects of glucagon on feeding. The biological relevance of this anorectic effect of glucagon is puzzling since glucagon increases during fasting and hypoglycemia, states that increase rather than decrease feeding. However, there are several diseases states, such as infection or chronic heart failure, in which elevated glucagon is associated with decreased food intake and cachexia [40]. In keeping with the anorectic action of glucagon as an activator of PKA, several lines of evidence suggest that hypothalamic PKA can produce anorectic effects [41], [42]. Therefore it is tempting to hypothesize that the anorectic effects of glucagon observed are of biological relevance in certain disease states, such as those mentioned above.

DIO animals exhibit an altered insulin/glucagon ratio and are resistant to the hypothalamic actions of glucagon on glucose production, suggesting that this resistance contributes to hyperglycemia in diabetes and obesity [8]. In line with those results, we also found a HFD-induced resistance to the anorectic action of glucagon. Our findings suggest that, in addition to the central resistance to certain hormones regulating food intake, such as leptin or insulin, central resistance to glucagon-induced hypophagia might also contribute to the development of obesity. Indeed, the next step was to search for factors of glucagon transduction signaling that might explain DIO-induced glucagon resistance. We found that although the central activation of PKA triggered pCREB levels, the amount of CaMKKβ and pAMPK in the hypothalamus of obese animals did not change. Thus, central glucagon was unable to decrease food intake in DIO rats, suggesting that glucagon signaling was disrupted downstream of PKA. These findings differ from a previous report indicating that the impaired hypothalamic glucagon action that lowers glucose production is upstream of PKA [8]. This, in turn, suggests that DIO-induced hypothalamic glucagon resistance that leads to hepatic glucose production or anorexia is mediated through different mechanisms.

Our next candidate to explain the obesity-induced hypothalamic resistance to the anorectic action of glucagon was CaMKKβ. Hypothalamic CaMKKβ is physiologically relevant for the regulation of energy balance. Mice lacking CaMKKβ are protected against HFD and show reduced hypothalamic AMPK activity and down-regulation of NPY and AgRP gene expression [43]. Using virogenetic tools to inhibit CaMKKβ activity within the ARC enabled us to restore the anorectic action of central glucagon in obese rats, suggesting that DIO-induced resistance to the anorexic function of glucagon can be explained by deficient CaMKKβ signaling.

In summary, we show that glucagon causes a rapid and transient decrease in food intake through a mechanism involving the glucagon receptor, PKA, CaMKKβ and AMPK within the ARC. This functional pathway is, however, blunted in DIO rats, suggesting a HFD-induced resistance to the anorectic action of central glucagon. The inhibition of CaMKKβ in the ARC is sufficient to restore glucagon sensitivity in obese animals. These data describe the molecular underpinnings by which glucagon controls feeding that may lead to a better understanding and therapeutic approaches of disease states linked to anorexia and cachexia.

## Figures and Tables

**Figure:**
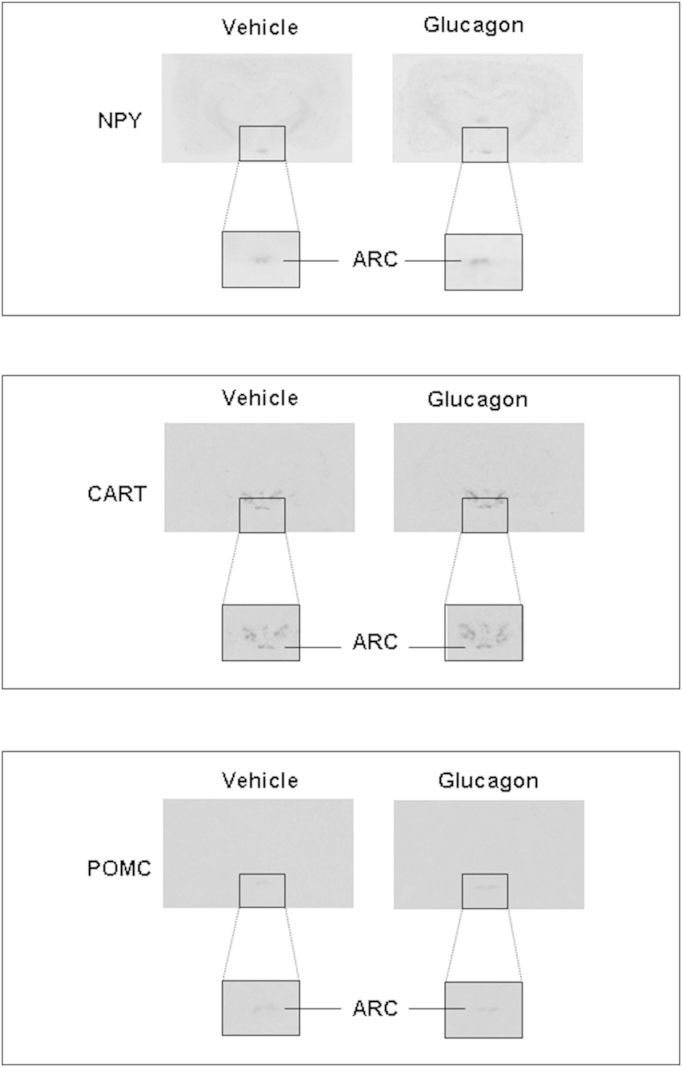


**Figure:**
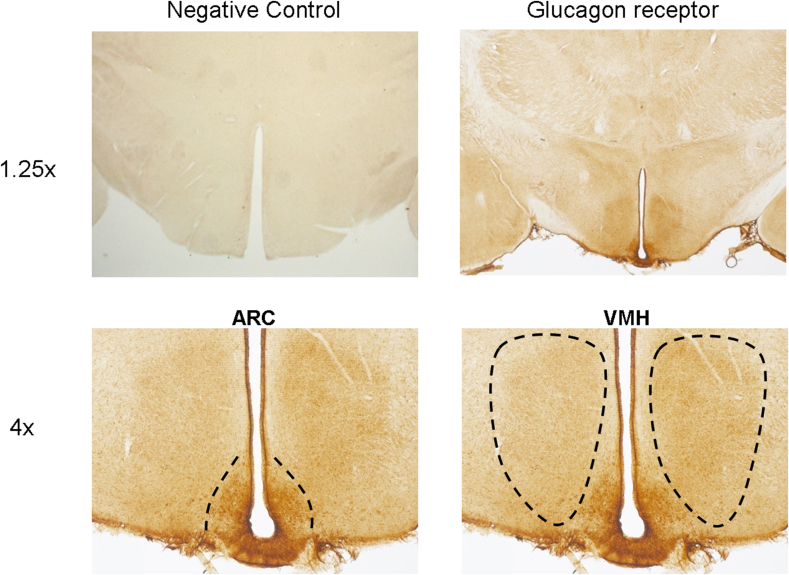


**Figure:**
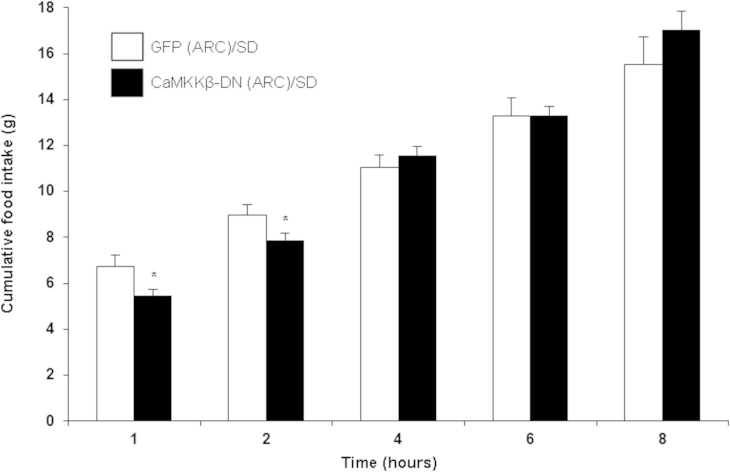


**Figure 1:**
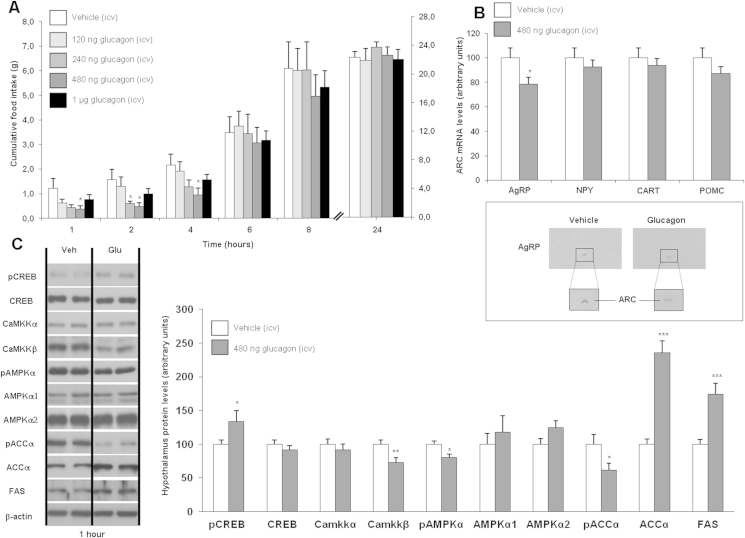
Effect of single ICV injections of glucagon (120, 240, 480 or 1000 ng/rat) on cumulative food intake (A), measured after 1, 2, 4, 6, 8 and 24 h. Effect of ICV glucagon (480 ng/rat) injections on neuropeptide mRNA expression (B) and protein levels (C) measured after 1 h. β-actin was used to normalize protein levels. Dividing lines indicate spliced bands from the same gel. Values are mean ± SEM of 7–8 animals per group. *P < 0.05; **P < 0.01; ***P < 0.001.

**Figure 2:**
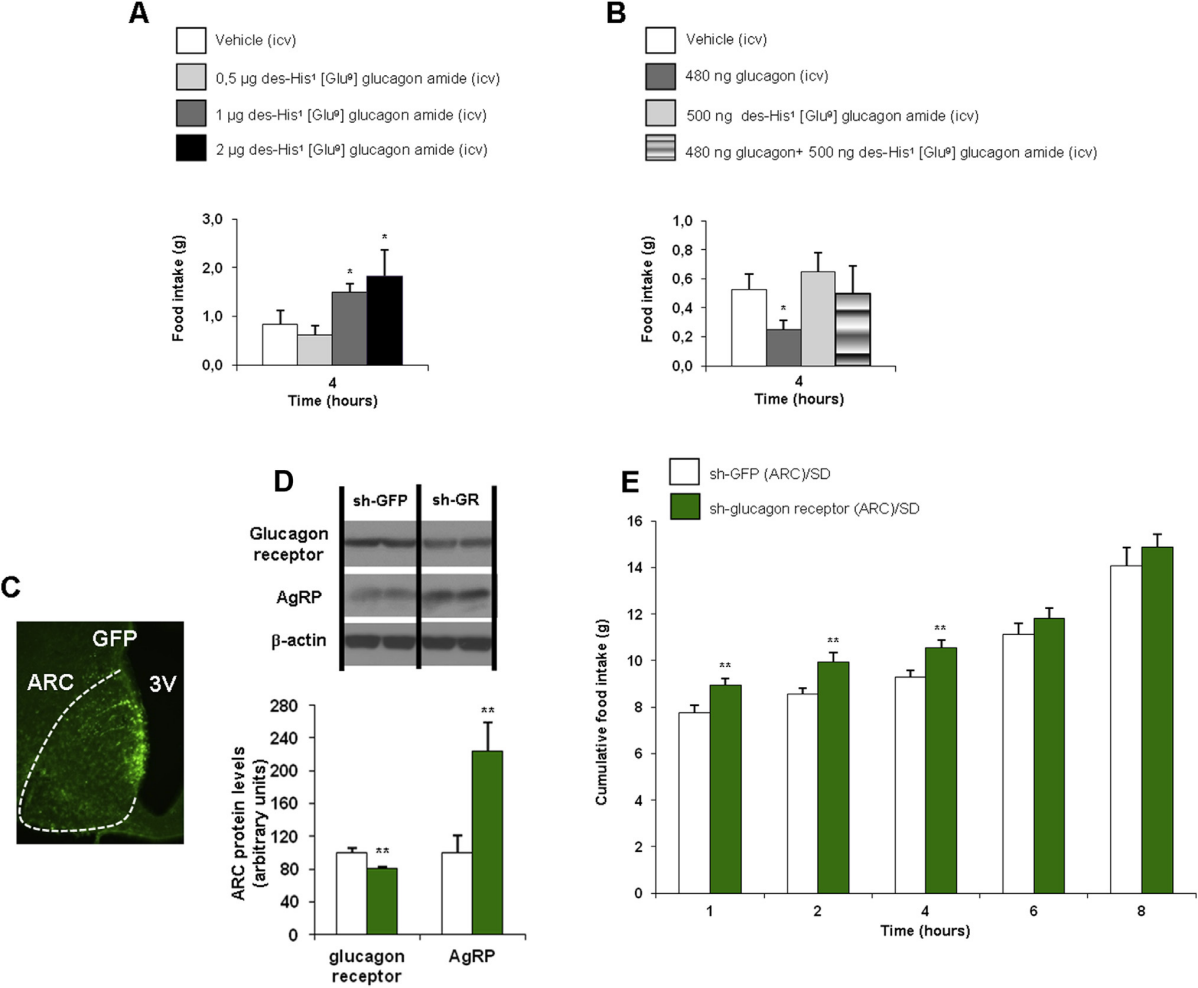
Effect of ICV glucagon receptor antagonist (des-His^1^ (Glu^9^) glucagon amide) (0.5, 1, and 2 μg/rat) on cumulative food intake, measured after 4 h (A). Food intake in rats injected ICV with vehicle, glucagon, glucagon receptor antagonist, and glucagon with a previous injection of the glucagon receptor antagonist (B). Effect of the injection of lentiviral particles encoding a green fluorescent protein (GFP) or the glucagon receptor in the ARC (C) and ARC protein levels of glucagon receptor (D). Cumulative food intake after overnight fasting (12 h) after the injection of lentiviruses encoding shRNA against glucagon receptor into the ARC of rats fed a chow diet (E). β-actin was used to normalize protein levels. Dividing lines indicate spliced bands from the same gel. Values are mean ± SEM of 7–8 animals per group. *P < 0.05; **P < 0.01.

**Figure 3:**
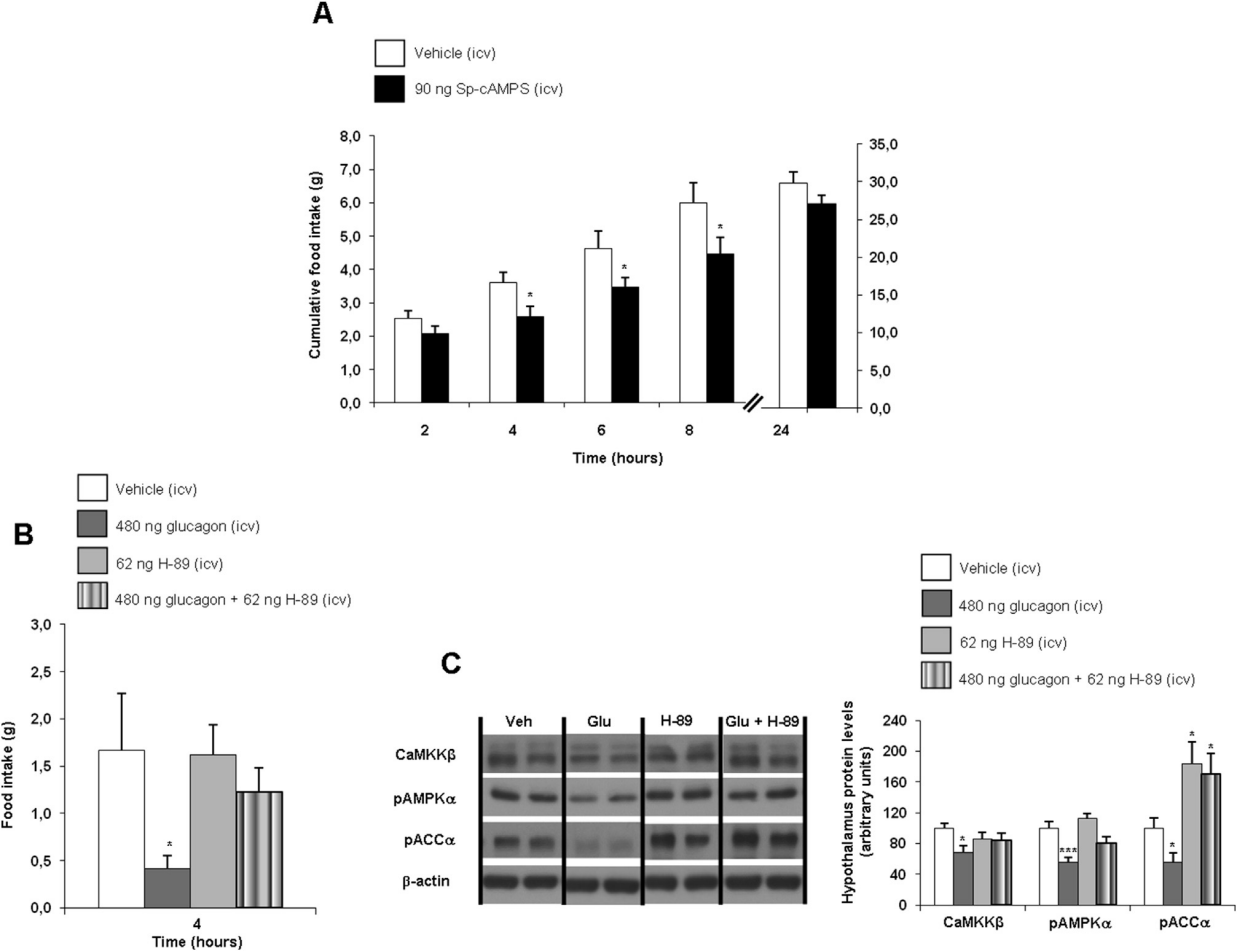
Effect of ICV vehicle and the PKA activator Sp-cAMPS (90 ng/rats) injections on cumulative food intake in rats fed a chow diet (A). Food intake (B) and hypothalamic protein levels (C) in rats fed a chow diet injected ICV with vehicle, glucagon, PKA inhibitor (H-89), and glucagon with a previous injection of H-89. β-actin was used to normalize protein levels. Dividing lines indicate spliced band from the same gel. Values are mean ± SEM of 7–8 animals per group. *P < 0.05; **P < 0.01; ***P < 0.001.

**Figure 4:**
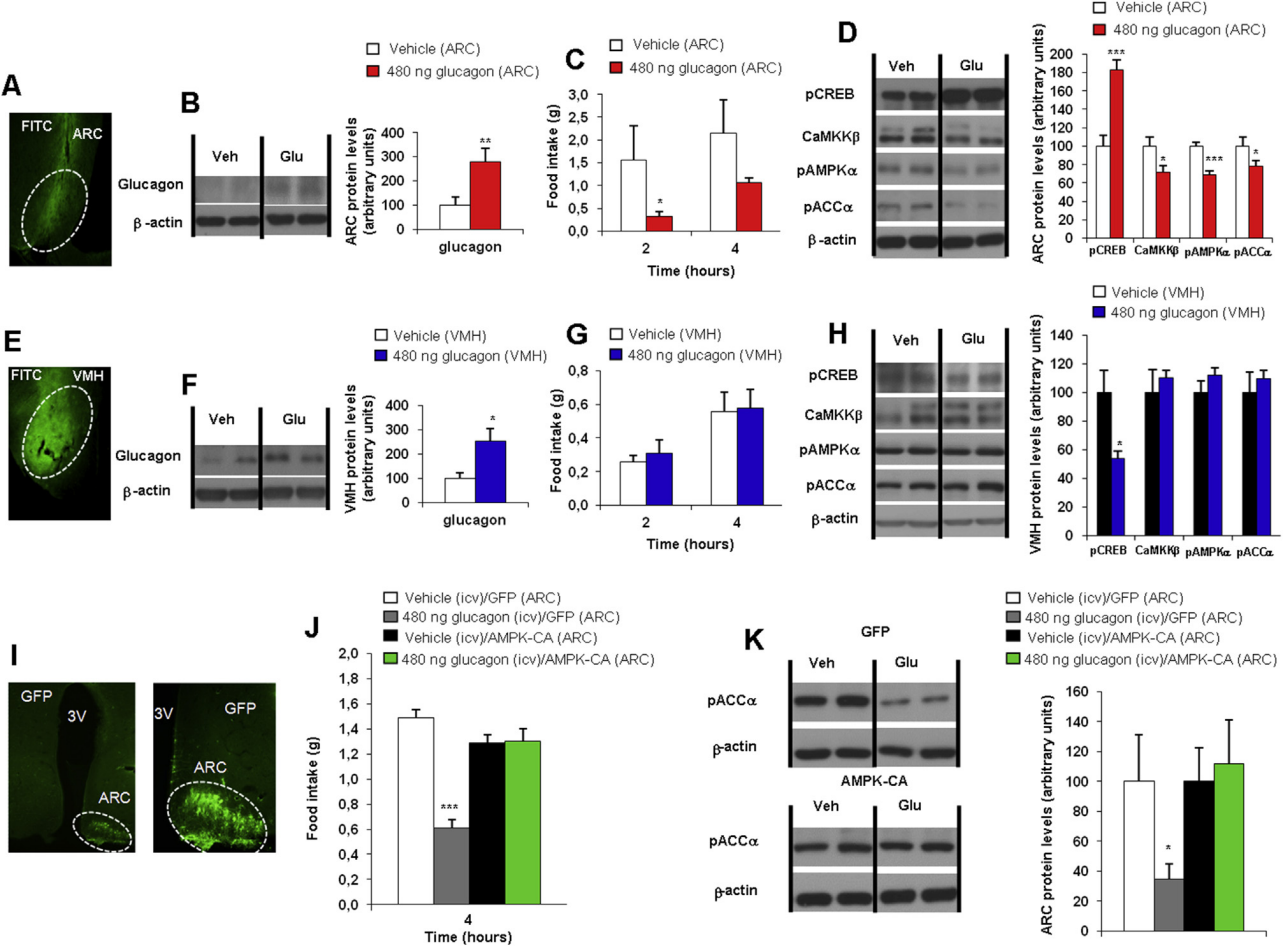
Fluorescein-isothio-cyanate (FITC) staining in the hypothalamic arcuate nucleus (ARC) (A), glucagon protein levels in the ARC (B), food intake (C) and ARC protein levels (D) in rats after injection of glucagon specifically in the ARC. FITC staining in the hypothalamic ventromedial nucleus (VMH) (E), glucagon protein levels in the VMH (F), food intake (G) and VMH protein levels (H) in rats after injection of glucagon specifically in the VMH. Effect of the injection of adenoviral particles encoding for a green fluorescent protein (GFP) or constitutively active AMPK (AMPKα-CA) in the ARC (I). Food intake (J) and ARC protein levels (K) were measured 1 h after ICV injection with glucagon. β-actin was used to normalize protein levels. Dividing lines indicate spliced bands from the same gel. Values are mean ± SEM of 7–8 animals per group. *P < 0.05; **P < 0.01; ***P < 0.001.

**Figure 5:**
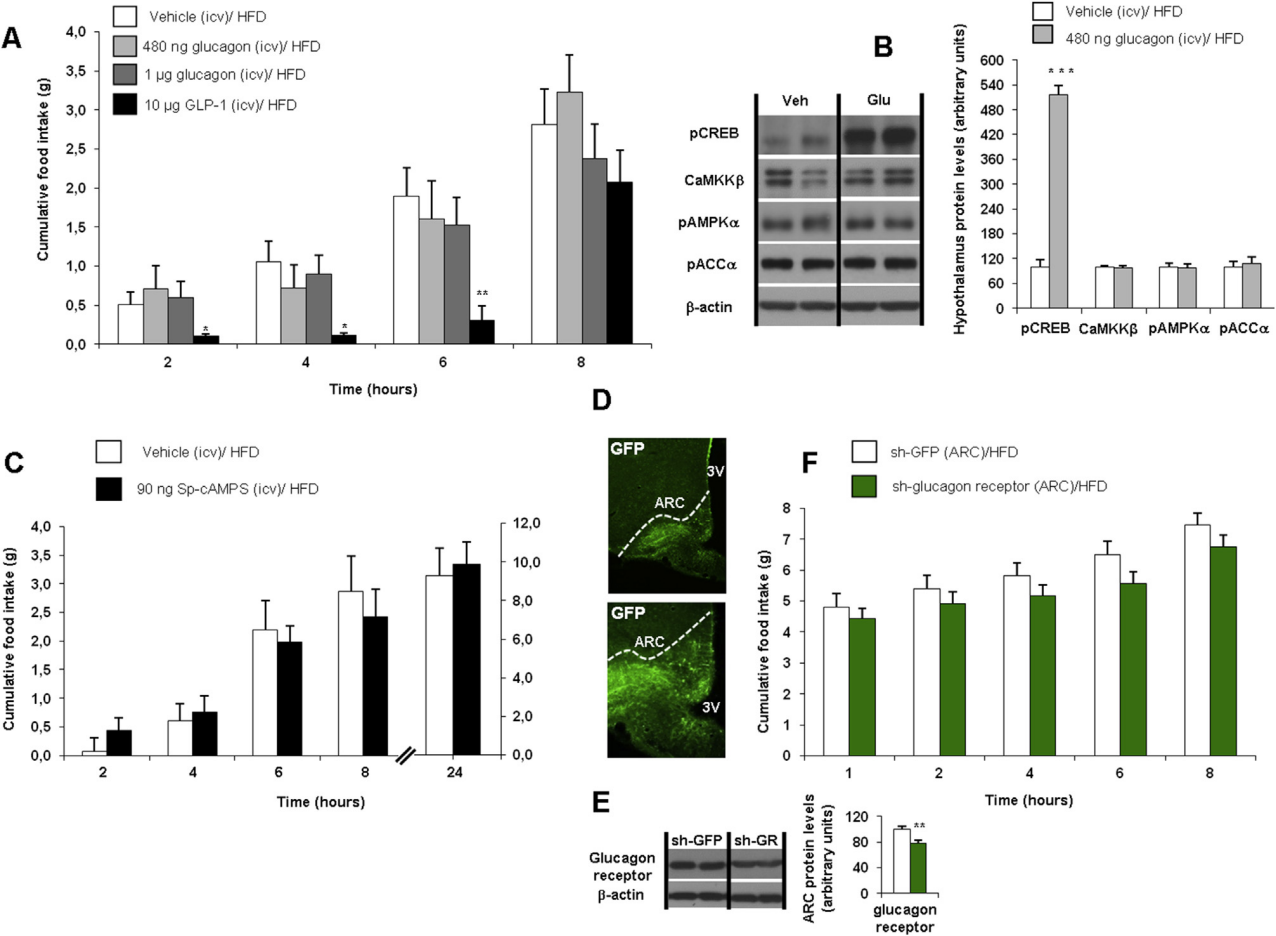
Effect of ICV glucagon (480, 1000 ng/rat) and GLP-1 (10 μg/rat) injections on cumulative food intake in rats fed a high fat diet (HFD) after 2, 4, 6 and 8 h (A). Effect of ICV glucagon (480 ng/rat) injection on hypothalamic protein levels after 1 h (B). Effect of ICV Sp-cAMPS (90 ng/rats) on cumulative food intake in rats fed a HFD after 2, 4, 6, 8 and 24 h (C). Effect of the injection of lentiviral particles encoding a green fluorescent protein (GFP) or the glucagon receptor in the ARC (D), glucagon protein levels in the ARC (E) and cumulative food intake after overnight fasting (12 h) after the injection of lentiviruses encoding shRNA against glucagon receptor into the ARC of rats fed a chow diet (F). β-actin was used to normalize protein levels. Dividing lines indicate spliced bands from the same gel. Values are mean ± SEM of 7–8 animals per group. *P < 0.05; **P < 0.01; ***P < 0.001.

**Figure 6:**
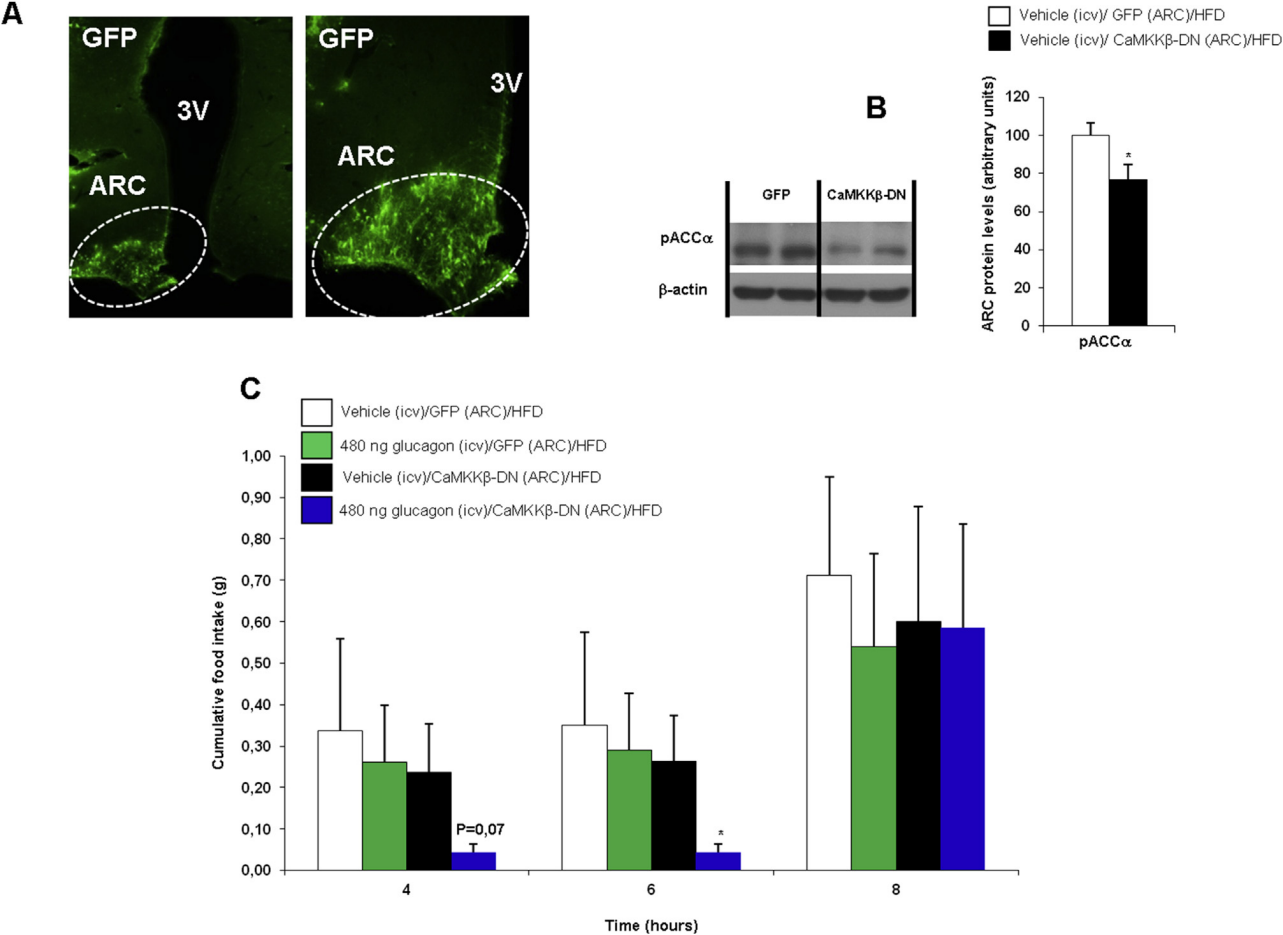
Effect of the injection of adenoviral particles encoding for a green fluorescent protein (GFP) or the dominant negative (DN) form of CAMKKβ in the ARC (A) and ARC protein levels of pACC (B). Effect of treatment with ICV glucagon on food intake in rats injected stereotaxically with CaMKKβ-DN adenoviruses into the ARC or injected stereotaxically with GFP adenoviruses into the ARC (C). β-actin was used to normalize protein levels. Dividing lines indicate spliced bands from the same gel. Values are mean ± SEM of 7–8 animals per group. *P < 0.05; **P < 0.01; ***P < 0.001.
