# Supplementary material for: Hypothalamic CaMKKβ mediates glucagon anorectic effect and its diet-induced resistance
Source: Mol Metab. 2015 Oct 22;4(12):961–70. doi: 10.1016/j.molmet.2015.09.014 (PMC4731730; doi:10.1016/j.molmet.2015.09.014)
Supplement: Supplementary file 1 [file mmc1.doc]

**Supplementary Material and Methods**

**Implantation of intracerebroventricular cannulae and treatments**

Intracerebroventricular cannulae were implanted stereotaxically in the lateral ventricle, in a stereotaxic frame (David Kopf Instruments, Tujunga, CA). The coordinates used were -0.9

mm posterior to bregma, ±1.6

mm lateral from midline and -3.4

mm ventral from the surface of the skull.

**Western blot analysis**

Protein lysates from total hypothalamus (30 μg), ARC (20 μg) and VMH (20 μg) were subjected to sodium dodecyl sulfate–polyacrylamide gel electrophoresis, electrotransferred on a polyvinylidene difluoride membrane, and probed with different antibodies (Supplementary table 1). For protein detection we used horseradish-peroxidase–conjugated secondary antibodies (Dako Denmark, Glostrup, Denmark) and chemiluminescence (Pierce ECL Western Blotting Substrate; Thermo Scientific, Waltham, MA). Then, the membranes were exposed to radiograph film (Super RX, Fuji Medical X-Ray Film; Fujifilm, Tokyo, Japan) and developed with developer and fixing liquids (AGFA, Mortsel, Belgium) under appropriate dark room conditions. The protein levels were normalized to β-actin for each sample.

***In situ* hybridization**

Coronal brain sections (16 mm) were probed with specific antisense oligos for agouti-related peptide (AgRP), neuropeptide Y (NPY), pro-opiomelanocortin (POMC) and cocaine- and amphetamine-regulated transcript (CART). Sections were scanned and the hybridization signal was quantified by densitometry using ImageJ-1.33 (NIH; Bethesda, MD, USA). We used between 16-20 sections for each animal (4-5 slides with four sections per slide). The mean of these 16-20 values was used as the densitometry value for each animal.

**Supplementary Table 1. Primary antibodies used for immunoblots**

| **Antibody** | **Vendor** | **Cat. No.** | **Fold Dilution** |
| --- | --- | --- | --- |
| Rabbit anti-ACCα  | Millipore | 04-322 | 1000 |
| Rabbit anti-AMPKα1 | Millipore | #07-350 | 1000 |
| Rabbit anti-AMPKα2 | Millipore | #07-363 | 1000 |
| Mouse anti-β-Actin | Sigma | A5316 | 5000 |
| Rabbit anti-CaMKKα (R-73) | Santa cruz biotechnology | sc-11370 | 1000 |
| Goat anti-CaMKKβ (L-19) | Santa cruz biotechnology | sc-9629 | 1000 |
| Rabbit anti-CREB1(240) | Santa cruz biotechnology | sc-58 | 1000 |
| Rabbit anti-FAS (H-300) | Santa cruz biotechnology | sc-20140 | 1000 |
| Rabbit anti-glucagon | Abcam | ab-92517 | 1000 |
| Rabbit anti-glucagon receptor | Abcam | ab-75240 | 1000 |
| Rabbit anti-phospho-ACCα (Ser79) | Millipore | #07-303 | 1000 |
| Rabbit anti-phospho-AMPKα (Thr172) | Cell Signaling Technology | #2535 | 1000 |
| Rabbit anti-phospho-CREB1 (Ser133) | Cell Signaling Technology | #9198 | 1000 |

**Supplementary Figures**

**Supplementary figure 1.** Effect of ICV glucagon (480 ng/rat) on NPY, CART and POMC mRNA expression. Values are mean ± SEM of 7-8 animals per group.

**Supplementary figure 2.** Immunohistochemistry showing glucagon receptor (1.25X upper panel and 4X lower panel) in the arcuate nucleus (ARC) and ventromedial nucleus (VMH).

**Supplementary figure 3.** Cumulative food intake after overnight fasting after the injection of CaMKKβ-DN adenoviruses into the ARC of rats fed a chow diet after 2, 4, 6 and 8 h.
